# Anti-infective Properties of the Golden Spice Curcumin

**DOI:** 10.3389/fmicb.2019.00912

**Published:** 2019-05-03

**Authors:** Dimas Praditya, Lisa Kirchhoff, Janina Brüning, Heni Rachmawati, Joerg Steinmann, Eike Steinmann

**Affiliations:** ^1^Department of Molecular and Medical Virology, Ruhr-University Bochum, Bochum, Germany; ^2^Institute of Experimental Virology, Twincore – Centre for Experimental and Clinical Infection Research, A Joint Venture Between the Medical School Hannover and The Helmholtz Centre for Infection Research, Hanover, Germany; ^3^Research Center for Biotechnology, Indonesian Institute of Science, Cibinong, Indonesia; ^4^Institute of Medical Microbiology, University Hospital Essen, University of Duisburg-Essen, Essen, Germany; ^5^School of Pharmacy, Bandung Institute of Technology, Bandung, Indonesia; ^6^Research Center for Nanosciences and Nanotechnology, Bandung Institute of Technology, Bandung, Indonesia; ^7^Institute of Clinical Hygiene, Medical Microbiology and Infectiology, Klinikum Nürnberg, Paracelsus Medical University, Nuremberg, Germany

**Keywords:** curcumin, natural products, nutraceutical, anti-infective properties, virus, bacteria, fungi

## Abstract

The search for novel anti-infectives is one of the most important challenges in natural product research, as diseases caused by bacteria, viruses, and fungi are influencing the human society all over the world. Natural compounds are a continuing source of novel anti-infectives. Accordingly, curcumin, has been used for centuries in Asian traditional medicine to treat various disorders. Numerous studies have shown that curcumin possesses a wide spectrum of biological and pharmacological properties, acting, for example, as anti-inflammatory, anti-angiogenic and anti-neoplastic, while no toxicity is associated with the compound. Recently, curcumin’s antiviral and antibacterial activity was investigated, and it was shown to act against various important human pathogens like the influenza virus, hepatitis C virus, HIV and strains of *Staphylococcus*, *Streptococcus*, and *Pseudomonas*. Despite the potency, curcumin has not yet been approved as a therapeutic antiviral agent. This review summarizes the current knowledge and future perspectives of the antiviral, antibacterial, and antifungal effects of curcumin.

## Introduction

Infectious diseases are ailments caused by pathogenic viruses and microorganisms such as bacteria and fungi. Infections can spread directly from person to person and from animal to human, or indirectly via contaminated water and food. This can result in small local outbreaks and epidemics, like the plague, syphilis and SARS, or pandemics affecting several countries, of which the flu is one of the best-known examples. In times of globalization and climate change, infectious diseases are spreading more rapidly than ever before, and new ones continue to emerge. Even though they are a global health burden, inhabitants of developing countries especially suffer from infections. Accordingly in 2010, worldwide, roughly one quarter of deaths was due to infectious diseases, while in low-income countries, nearly 60% of fatalities could be attributed to them (Dye, 2015). This is primarily because in these regions often hygienic measures are insufficient, diagnostic tools are lacking and therapeutic options are not available.

Existing medications are categorized into antivirals used to combat viral diseases, antibiotics contradicting bacterial infections and antifungals inhibiting the growth of fungi. In addition, multiple vaccines preventing viral and bacterial diseases exist, which has already led to the successful eradication of smallpox. However, countermeasures are available only for a limited number of pathogens, not including all potentially lethal and pandemic agents, as e.g., Ebola virus, and resistance to current therapies is increasing. Thus, new therapeutic options are urgently needed. Natural compounds are a continuing source of new drugs. From 1940 to 2014, 49% of all small molecules approved by the US Food and Drug Administration (FDA) were natural products or derivates directly linked to them (Newman and Cragg, 2016). One plant that has been extensively studied on that score is turmeric.

**FIGURE 1:**
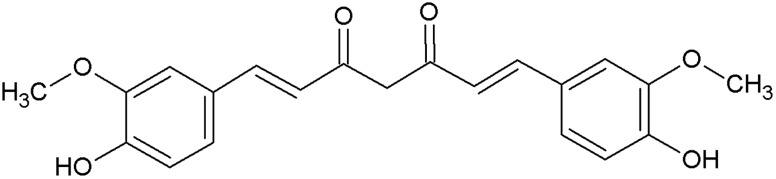
Chemical structure of curcumin.

Turmeric (*Curcuma longa* L.) belongs to the family of ginger (Zingiberaceae) and natively grows in India and Southeast Asia. The plants rhizomes contain several secondary metabolites including curcuminoids, sesquiterpenes, and steroids (Omosa et al., 2017); with the curcuminoid curcumin being the principal component of the yellow pigment and the major bioactive substance. Chemically, curcumin is a diferuloylmethane, a diarylheptanoid belonging to the class of natural phenols. Its chemical structure has been described already in 1910 as a symmetric molecule of two phenol rings connected by α,β-unsaturated carbonyl groups(Miłobȩdzka et al., 1910) (see Figure 1).

In Europe, curcumin is widely used as a dye to color products in a bright to golden yellow. Historically, it was utilized mostly for leather and fabric, while nowadays, it functions as a food coloring. In the European Union the molecule is approved as a food additive and can be found labeled as E100 in the ingredient list of many groceries, including baked goods, sweets, spreads, or cheese. In the Asian society, ground turmeric has been used as a spice for centuries. It also plays a role in traditional Chinese and Indian medicine, where it is used to treat different maladies such as dermatologic ailments, infections, liver complaints, and depression. The use of curcumin is not associated with toxicity, and the FDA categorized it as “Generally Recognized As Safe.” Thus, the medical properties of the natural product have been widely investigated. Today, a literature search at pubmed.gov finds over 11,000 publications, while a quest at clinicaltrials.gov reveals 179 clinical studies using curcumin. Most studies analyzed curcumin’s anti-cancer effect and it has been shown to inhibit tumor cell proliferation, invasion and metastatic dissemination (as reviewed, e.g., Bachmeier et al., 2018). Besides this, curcumin has been documented to act, e.g., anti-inflammatory and anti-infective (as reviewed, e.g., Hatcher et al., 2008) and due to its wide spectrum of biological and pharmacological properties it is often called “cure-cumin.”

In this review, we will give an up-to-date overview of the anti-infective properties of curcumin. At first, we will summarize the antiviral effect of the molecule against different virus families. We will then reflect the antibacterial and the antifungal activities of the compound. Finally, we will discuss the obstacles and the future perspectives regarding the use of curcumin as a therapeutic drug.

## Antiviral Activities of Curcumin

Due to the lack of preventive and therapeutic options for many viral infections, numerous studies have been conducted to investigate the antiviral potential of natural compounds. Accordingly, antiviral effects haven been shown, e.g., for components of green tea (as reviewed, e.g., Steinmann et al., 2013), cinnamon (Connell et al., 2016) and many herbs. For curcumin, an antiviral activity was observed against several different viruses including hepatitis viruses, influenza viruses and emerging arboviruses like the Zika virus (ZIKV) or chikungunya virus (CHIKV). Interestingly, it has also been reported that the molecule inhibits human immunodeficiency virus (HIV), herpes simplex virus 2 (HSV-2) and human papillomavirus (HPV), indicating that curcumin reduces the spread of sexually transmitted diseases. In this section and in Table 1 we will summarize the current understanding of the antiviral aspects of curcumin and possible mechanisms underlying its inhibitory effects.

**Table 1:** Antiviral activity of curcumin against several different viruses.

Virus	Family	Antiviral activity	References
CHIKV	*Togaviridae*	Entry inhibitor	Rhein et al., 2016
DENV	*Flaviviridae*	Entry inhibitor Particle production inhibition	Chen et al., 2013 Padilla-S et al., 2014
HBV	*Hepadnaviridae*	Replication inhibitor cccDNA inhibitor	Kim et al., 2009, 2011; Rechtman et al., 2010 Wei et al., 2017
HCV	*Flaviviridae*	Entry inhibitor	Anggakusuma et al., 2014
HIV	*Retroviridae*	Protease inhibitor Integrase inhibitor Tat protein inhibitor	Sui et al., 1993 Mazumder et al., 1995 Barthelemy et al., 1998; Balasubramanyam et al., 2004; Ali and Banerjea, 2016
HPV	*Papilomaviridae*	Gene expression inhibition	Maher et al., 2011; Mishra et al., 2015
HSV	*Herpesviridae*	Gene expression inhibition	Kutluay et al., 2008
IAV	*Orthomyxoviridae*	Entry inhibitor	Chen et al., 2010; Ou et al., 2013
JEV	*Flaviviridae*	Entry inhibitor Particle production inhibition	Chen et al., 2013 Padilla-S et al., 2014
MNV	*Caliciviridae*	Entry inhibitor	Yang M. et al., 2016
RSV	*Pneumoviridae*	Entry inhibitor Replication and budding inhibition	Yang X.X. et al., 2016; Yang et al., 2017 Obata et al., 2013
RVFV	*Phenuiviridae*	Replication inhibitor	Narayanan et al., 2012
ZIKV	*Flaviviridae*	Entry inhibitor	Mounce et al., 2017


### Curcumin Inhibits Human Immunodeficiency Virus

The HIV is a lentivirus which belongs to the family of *Retroviridae*. HIV is the causative agent of the acquired immunodeficiency syndrome, better known as AIDS, and since its first report in 1981 it has become one of the major global public health issues: worldwide, more than 35 million people are infected with HIV, and in 2017 approximately 0.95 million people died of its consequences (WHO, 2018). Still, to date no preventive vaccine or effective cure exists for HIV.

Several studies have reported that curcumin exhibits an anti-HIV activity by directly targeting viral proteins. Already in 1993, Sui et al. reported a modest inhibition of the HIV-1 and HIV-2 proteases by curcumin. The authors found that the molecule binds to multiple sites of the enzyme, with micro molar concentrations leading to a suppression of enzymatic activity. A curcumin-boron complex showed an improved inhibitory effect, which is due to the complex’s binding to additional sites within the substrate-binding cavity of the protease. In line with this, *in silico* modeling studies confirmed that curcumin fits well to the protease active site (Vajragupta et al., 2005). Besides the protease also the HIV integrase is an interesting drug target, because the enzyme is responsible for the integration of the viral genome into the host DNA. It has been shown that curcumin is a potent HIV integrase inhibitor, as it is able to bind acidic residues in the integrases catalytic core domain, preventing it from binding its substrates (Mazumder et al., 1995). Computational docking studies revealed that specifically the keto-enol and terminal o-hydroxyl group of curcumin exhibit tight linkage to the integrases binding site formed by residues Asp64, His67, Thr66, Glu92, Thr93, Asp116, Ser119, Asn120, and Lys159 (Vajragupta et al., 2005). Another HIV protein targeted by curcumin is the trans-activator of transcription (tat), a viral transcription regulator. Upon infection, tat is secreted and taken up by uninfected cells, which promotes the growth of HIV-induced tumors and the apoptosis of T-cells, fostering the development of AIDS (Ensoli et al., 1990; Westendorp et al., 1995; Campbell et al., 2004). Thus, inhibiting tat would prevent efficient viral gene transcription as well as disease progress. Interestingly, tat is known to be an intrinsically disordered protein (Shojania and O’Neil, 2010) and curcumin is known to induce the degradation of proteins with partially intrinsically disordered regions, like p53 (Tsvetkov et al., 2005). Accordingly, in tat-transduced HEK293T cells the tat protein level decreased upon incubation with curcumin in a dose-dependent manner that can be blocked by proteasome inhibitors, indicating that curcumin causes proteasomal degradation of tat (Ali and Banerjea, 2016). Moreover, it has been reported that curcumin inhibits HIV proliferation by inhibiting tat acetylation in SupT1 cells (Balasubramanyam et al., 2004) and that curcumin efficiently inhibits tat-induced transactivation of HIV-1 long terminal repeats in HeLa cells (Barthelemy et al., 1998).

In addition to targeting viral proteins, curcumin has also been described to indirectly inhibit HIV or the progression of AIDS by reducing HIV-induced cancer, inflammation and others. Despite this, a clinical study conducted in the 1990s could not find a decreased viral load or increased T-cell count in 40 HIV-patients treated with the molecule for 8 weeks (Gilden and Smart, 1996; James, 1996). Recently, new curcumin derivates or formulations with increased bioavailability and stability were developed against HIV (Kumari et al., 2015; Lin et al., 2017; Sharma et al., 2017; Zhao et al., 2017) and it has been shown that curcumin pretreatment of human genital epithelial cells blocks the infection-mediated induction of chemokines associated with the recruitment of HIV-target cells (Ferreira et al., 2015). Clinical assessment will show, whether these new curcumin variants and mechanisms of protection are effective in humans. Concluding, the attempt to use curcumin as an anti-HIV drug is still ongoing.

### Curcumin Inhibits Hepatitis Viruses

Viral hepatitis is one of the major causes of chronic liver disease, leading to more deaths than those caused by HIV, according to the World Health Organization (WHO) (WHO, 2017). In 2015 worldwide 256 million people were chronically infected with the hepatitis B virus (HBV; *Hepadnaviridae*) (WHO, 2017) and 71 million people suffered from chronic hepatitis C virus (HCV; *Flaviviridae*) infections (WHO, 2017). In addition, sporadic hepatitis A virus (*Picornaviridae*) and hepatitis E virus (*Hepeviridae*) outbreaks occur, affecting approximately 14 and 20 million people every year, respectively (Rein et al., 2012; Percivalle et al., 2016). To date, a globally licensed vaccine is only present for HBV, while curative medications are only available for HCV. Besides developing new therapeutic options, also raising the awareness of transmission risks and reducing virus spread are important steps in controlling these infections (as reviewed, e.g., Pfaender et al., 2016).

The antiviral effect of curcumin against hepatitis viruses has been investigated by several groups. First hints that curcumin acts on HBV were given by studies suggesting that aqueous extracts of *Curcuma longa* inhibited the production of HBV particles and operated beneficial on the development of HBx protein-induced hepatocellular carcinoma (Kim et al., 2009, 2011). Further research revealed that indeed curcumin inhibits HBV gene expression and replication by down-regulating PGC-1a, a protein co-activating HBV transcription (Rechtman et al., 2010). A recent study even indicated that curcumin reduces the presence of covalently closed circular HBV DNA (cccDNA) *in vitro* (Wei et al., 2017), a holy grail in the cure of chronic HBV.

Curcumin was also found to be effective against HCV without cytotoxicity. Anggakusuma et al. (2014) showed that curcumin inhibits the entry of all HCV genotypes into hepatoma cells and primary human hepatocytes. The authors found that curcumin, especially its α,β-unsaturated ketones, affects the fluidity of the viral envelope and by this impairs its binding and fusion with the plasma membrane during cell entry and cell-to-cell spread. These findings are supported by earlier studies, which illustrated that curcumin changes the lipid bilayer properties of membranes, making them less stiff and leading to the disruption of liposomes (Ingolfsson et al., 2007; Chen et al., 2013). Besides entry, also later steps in the HCV life cycle have been reported to be impaired by curcumin. Accordingly, several studies showed that curcumin reduces HCV replication, but the exact mode of action is still unclear (Kim et al., 2010; Chen et al., 2012). Combination studies demonstrated that curcumin even acts synergistic with (clinically used) HCV inhibitors as interferon-α, Boceprevir and Cyclosporin (Kim et al., 2010; Anggakusuma et al., 2014) however, it’s low bioavailability leads to no therapeutic effect *in vivo* (Anggakusuma et al., 2014).

### Curcumin Inhibits Influenza A Virus

Influenza viruses belong to the family *Orthomyxoviridae* and can be divided into three types: A, B, and C. The influenza A virus (IAV) mainly infects birds, but it can cause severe pandemics in domestic poultry and in humans, even though this happens rarely. Currently two classes of drugs are FDA-approved for the treatment of influenza: matrix protein 2 (M2) inhibitors (amantadine, rimantadine) and neuraminidase inhibitors (oseltamivir, zanamivir, and peramivir). However, the emergence of drug-resistant viruses continuously increases, thus the use of M2-inhibitors is not recommended anymore. Therefore, new antiviral targets with novel inhibition mechanisms are urgently needed.

Several studies tested the effect of curcumin on different IAV types *in vitro* and found it to inhibit virus uptake, replication and particle production (Chen et al., 2010; Dai et al., 2018; Han et al., 2018). Experimental work and structure-relationship modeling suggested that the inhibition was due to the molecule interfering with binding of the viral glycoprotein hemagglutinin (HA) to its cellular receptor (Chen et al., 2010; Ou et al., 2013). A subsequent study confirmed this effect and explained it by curcumin’s ability to modulate the features of lipid bilayers (Chen et al., 2013). Interestingly, curcumin’s structural analog monoacetyl-curcumin seems not to act on HA binding, but on Akt phosphorylation required for IAV propagation (Richart et al., 2018). The compound alone was as effective in dampening IAV infection as pure curcumin and a synergistic effect of the two analogs was observed.

Besides acting directly antiviral, recent *in vivo* studies showed that curcumin treatment reduces lung inflammation due to IAV infection in mice and increases the immune response toward IAV in turkeys (Umar et al., 2016; Han et al., 2018). Thus, curcumin treatment could be an alternative strategy to combat IAV infections and its sequelae.

### Curcumin Inhibits Herpesviruses

The family of *Herpesviridae* consists of many DNA viruses causing persistent, latent infections with no curative therapy present to date. Several members of the family show a very high prevalence in humans worldwide.

The most famous herpesvirus is probably the herpes simplex virus, which is categorized into two types: HSV-1, commonly associated with orofacial ulcer and HSV-2, which mainly causes genital ulcers. In 2012, the WHO estimated the global incidence of HSV-1 and HSV-2 infections to be 67 and 11%, respectively (Looker et al., 2015). Several studies found that low micro molar, not cytotoxic amounts of curcumin dampened HSV-1 and HSV-2 infectivity *in vitro* and *in vivo* (Bourne et al., 1999; Kutluay et al., 2008; Zandi et al., 2010). For HSV-1 this is associated with a considerably decreased expression of viral immediate early (IE) genes, which is due to a reduced RNA polymerase II recruitment to IE gene promoters (Kutluay et al., 2008). Intriguingly, similar to HIV, the pretreatment of human genital epithelial cells with the substance reduced the shedding of HSV-2 from these cells, a mechanism that might help to stop the spread of the sexually transmitted disease (Ferreira et al., 2015). Another highly prevalent *Herpesviridae* member is the human cytomegalovirus (HCMV). As shown for HSV-1, curcumin leads to a decreased IE gene expression during HCMV infections (Lv et al., 2014). This is probably caused by curcumin downregulating the cellular heat shock protein 90, a protein needed for HCMV IE gene expression (Lv et al., 2015).

Besides HSV and HCMV, curcumin has also been suggested to influence Epstein–Barr virus (EBV) infections. However, its effect is unclear, as one study reported the molecule to inhibit the reactivation of EBV (Hergenhahn et al., 2002), while another study showed that curcuminoids enhance lytic reactivation of the virus in nasopharyngeal and gastric carcinomas (Ramayanti et al., 2018).

### Curcumin Inhibits Human Papillomavirus

The *Papilomaviridae* family includes small, non-enveloped DNA viruses of which more than 150 different genotypes have been identified in humans. Human papillomaviruses cause persistent cutaneous or mucosal infections, and the infection with at least 13 HPV types is associated with the development of multiple types of cancer. Especially the incidence of cervical cancer, the fourth-most common cancer in women, is tightly linked to HPV infection (WHO, 2014) and in over 60% of the cases the high-risk HPV types 16 and 18 are detected (Clifford et al., 2003).

The effect of curcumin on HPV infection and HPV-associated tumor growth has been extensively studied (as reviewed, e.g., Teymouri et al., 2017). Already in 1990 the group of Howley could show that the viral oncoprotein E6 of HVP-16 and 18 complexes with the tumor suppressor protein p53 (Scheffner et al., 1990), targeting it for proteasomal degradation (Scheffner et al., 1990). *In silico* modeling suggested that curcumin binds to the p53 interaction site of E6, by this prohibiting it from binding p53 (Mamgain et al., 2015). In addition, *in vitro* studies showed that curcumin inhibited E6 and E7 expression and by this rescued p53 levels (Maher et al., 2011; Mishra et al., 2015). Several groups then developed creams and capsules containing curcumin for the local application to prevent or treat HPV infections, by this circumventing curcumin’s low bioavailability problem. These creams indeed suppressed the growth of HPV-positive cells and cervical tumors and even induced apoptosis of cervical cancer cells *in vitro* and *in vivo* (Singh and Singh, 2009; Debata et al., 2013; Mukherjee et al., 2017). Clinical studies confirmed that a topical, cervical application of curcumin had no toxic effect on healthy women and indicated that it led to an increased HPV clearance rate (Basu et al., 2013; Gattoc et al., 2017). Thus, curcumin formulations could potentially be used to prevent sexually-transmitted HPV infections or to treat cervical dysplasia caused by the virus.

### Curcumin Inhibits Respiratory Syncytial Virus

The human respiratory syncytial virus (RSV) causes respiratory tract infections and is one of the leading causes of morbidity and mortality in children under 5 years of age. In 2015, it has been estimated to have caused 33.1 million episodes of acute lower respiratory infection and an overall mortality of 118.200 deaths globally (Shi et al., 2017). To date, the broad-spectrum antiviral ribavirin is the only approved countermeasure for severe RSV infection, but clinical trials could not proof its efficacy (Law et al., 1997; Long et al., 1997). Due to the lack of effective RSV treatment and vaccine, there is an urgent need for novel antivirals.

The use of curcumin against RSV infections revealed that it prevented RSV replication and budding from human nasal epithelial cells and at the same time increased the epithelial barrier function, while it did not affect RSV in lung cells (Obata et al., 2013). To enable a local application of curcumin, Yang and coworkers just recently developed two different types of nanomaterials loaded with the compound, which showed good biocompatibility and abrogated RSV infection (Yang X.X. et al., 2016; Yang et al., 2017). The nanoparticles seemed to directly bind RSV, inhibiting virus–host interactions and leading to a significant decrease of infected cells. Future studies will show, whether the use of curcumin loaded nanoparticles is applicable and effective *in vivo*.

### Curcumin Inhibits Noroviruses

Noroviruses are members of the *Caliciviridae* family, which commonly cause acute gastroenteritis in developed as well as in developing countries. The WHO ranks human noroviruses (NoV) as the number one cause of foodborne illness and the number four cause of foodborne deaths globally (WHO, 2015). Today, the treatment of NoV infections is only symptomatic, and the focus is more on preventing the disease and its expansion. While incubation of murine norovirus (MNV) with curcumin was found to significantly neutralize subsequent infections of a mouse macrophage cell line in a time- and dose-dependent manner, it had no effect on a NoV-replicon carrying hepatoma cell line (Yang M. et al., 2016). This indicates, that curcumin acts on early steps in the viral life cycle before replication. Further studies are needed to clarify whether curcumin could be used as an anti-NoV therapy option. Different studies analyzed the potential of photodynamic therapy (PDT) with curcumin as a photosensitizer to prevent NoV transmission through contaminated foods. PDT is a technique often used in cancer studies, which uses light in combination with a photosensitizing molecule to elicit cell death due to the production of reactive oxygen species (ROSs). It was shown that the use of curcumin together with LED light significantly reduced MNV titers in buffer and in oysters (Wu et al., 2015; Randazzo et al., 2016), probably by destroying genome integrity and viral capsid protein stability (Wu et al., 2015). It still needs to be elucidated whether this also works for NoV and whether curcumin-PDT might be a novel approach to avoid norovirus transmission in the food-industry.

### Curcumin Inhibits Arboviruses

The group of arboviruses comprises different viruses which are transmitted by arthropod vectors like ticks and mosquitos. Nowadays arboviruses are rapidly re-emerging worldwide, as arthropod habitats are expanding due to climate change and the increase of global traffic. For most arboviruses, except Japanese encephalitis virus (JEV; *Flaviviridae*) and yellow fever virus (*Flaviviridae*), no vaccines are available and there is a lack of specific preventing or curing treatment for all of them.

Recently, two studies revealed that curcumin blocks the entry of CHIKV (*Tongaviridae*) by inhibiting its binding to host cells (Rhein et al., 2016; Mounce et al., 2017). Incubation of cells with the natural compound also significantly dampened infection with Dengue virus (DENV; *Flaviviridae*), JEV and ZIKV (*Flaviviridae*) via the same mechanism (Chen et al., 2013; Mounce et al., 2017). Similar as for HCV and IAV, this might be due to curcumin’s influence on membrane properties (Ingolfsson et al., 2007; Chen et al., 2013). In addition to inhibiting virus entry, curcumin treatment of cells already infected with DENV or JEV resulted in the intracellular accumulation of viral proteins and a reduction of viral particle production (Dutta et al., 2009; Padilla-S et al., 2014). Also Rift Valley fever virus (RVFV; *Phenuiviridae*) has been shown to be inhibited by curcumin: Narayanan et al. (2012) observed that the molecule inhibits IKK-mediated phosphorylation of the viral NSs protein, resulting in reduced viral replication. Notably, this did not only hold true *in vitro*, but also mice subcutaneously treated with curcumin showed an increased survival (60% compared to untreated animals) and a decreased hepatic viral load (90% compared to controls).

Interestingly, curcumin seems to not only act antiviral on several arboviruses, but might also be useful for reducing the spread of arthropod vectors: dietary uptake of essential oils from turmeric led to modest lethality in larvae and pupae of *Aedes aegypti*, the mosquito transmitting, e.g., CHIKV, DENV, YFV, and ZIKV (Kalaivani et al., 2012).

## Antibacterial Activities of Curcumin

Bacterial infections can cause a number of human diseases, including relatively harmless self-limiting ailments and potentially lethal medical conditions if left untreated. Potent antibiotics are available against many bacteria. Nevertheless, due to the extensive use of the drugs, antibiotic resistance is on the rise, making formerly easy to eliminate pathogens untreatable. As for other infectious agents globalization has contributed to the expansion of (resistant) strains. In response to this, in 2017 the WHO published a list of 12 bacterial strains against which new drugs are critically needed (Tacconelli et al., 2018). Among them are strains of *Staphylococcus, Streptococcus, Helicobacter* and *Pseudomonas*, which all have been shown to be inhibited by curcumin. In the following chapter, we will summarize today’s research status of curcumin’s activity against Gram-positive and Gram-negative bacteria, as also given in Table 2.

**Table 2:** Antibacterial activity of curcumin.

Bacteria	Antibacterial activity	References
*Staphylococcus aureus*	Growth inhibition Sortase A inhibitor	Bhawana et al., 2011; Krausz et al., 2015 Park et al., 2005
*Staphylococcus epidermidis*	Growth inhibition Biofilm formation inhibition	Tajbakhsh et al., 2008; Liu and Huang, 2012 Hegge et al., 2012
*Streptococcus* *mutans*	Adhesion inhibition Biofilm formation inhibition Sortase A inhibitor	Song et al., 2012 Li et al., 2018 Hu et al., 2013
*Streptococcus* *pyogenes*	Growth inhibition	Betts et al., 2016
*Bacillus subtilis*	Growth inhibition FtsZ inhibitor	Rai et al., 2008; Wang et al., 2009; Bhawana et al., 2011; Jaiswal and Mishra, 2018 Rai et al., 2008
*Bacillus cereus*	Growth inhibition	Rai et al., 2008; Wang et al., 2009; Bhawana et al., 2011; Jaiswal and Mishra, 2018
*Listeria innocua*	Growth inhibition	de Oliveira et al., 2018
*Escherichia coli*	Growth inhibition Biofilm formation inhibition	Wang et al., 2009; Bhawana et al., 2011; Jaiswal and Mishra, 2018
*Salmonella enteritica* serotype Typhimurium	Growth inhibition	Tønnesen et al., 1987; Dahl et al., 1989
*Helicobacter pylori*	Growth inhibition	De et al., 2009
*Pseudomonas aeruginosa*	Growth inhibition Biofilm formation inhibition	Bhawana et al., 2011; Krausz et al., 2015; Jaiswal and Mishra, 2018


### Curcumin Inhibits Staphylococci

*Staphylococcus aureus* is known to be among the most common pathogens causing both community and hospital acquired infections and it is the most important causative agent of bloodstream bacterial infections worldwide (Biedenbach et al., 2004).

Infections with methicillin-resistant *S. aureus* (MRSA), a type of *Staphylococcus* being resistant to certain antibiotics as β-lactams, are more difficult to treat. This resistance is based on the lowered β-lactam affinity of penicillin binding proteins encoded by mecA. Thus, infections with MRSA are characterized by a high mortality rate and increased health care costs (Cosgrove et al., 2003). MRSA was first reported in 1961, only 2 years after the introduction of methicillin against penicillin-resistant *S. aureus* (Jevons, 1961). Today, *S. aureus* including MRSA is endemic and among the deadliest pathogens (Klevens et al., 2007).

The activity of curcumin against staphylococci has been assessed in several studies. *In vitro* data revealed antimicrobial activity of curcumin against both, MRSA and methicillin-sensitive *S. aureus* (MSSA), with determined minimum inhibitory concentrations (MICs) in the micro molar range (Tajbakhsh et al., 2008; Mun et al., 2013; Teow and Ali, 2015; Jaiswal and Mishra, 2018). Furthermore, a synergistic effect of curcumin and different antibiotics (oxacillin, ampicillin, ciprofloxacin, gentamicin, amikacin, polymyxin B, and norfloxacin) was detected in a strain dependent manner, while no antagonistic effects were observed (Mun et al., 2013; Teow and Ali, 2015; Betts et al., 2016). The synergistic effects might possibly occur due to the capability of curcumin to bind bacterial enzymes, reducing lysis and hydrolyzation of antibiotics (Zhou et al., 2011; Teow and Ali, 2015).

Curcumin is known to be a relatively instable molecule with a particle size of 500–800 nm, impairing cellular uptake and resulting in low bioavailability (Wang et al., 2009; Bhawana et al., 2011). Various methods to improve its stability and bioavailability were investigated. Wang and colleagues used a capsulation technique to stabilize curcumin. They described a MIC of microencapsulated curcumin of 62.5 μg/ml against *S. aureus*, which is much lower compared to pure curcumin (Wang et al., 2009). Another approach is the construction of nanoparticles loaded with curcumin. Most recently, Jaiswal and Mishra compared the MICs of curcumin and curcumin-silver nanoparticles, revealing treatment with curcumin-silver nanoparticles being more effective against *S. aureus* (MIC = 5 μg/ml) (Jaiswal and Mishra, 2018). Nanocurcumin was previously shown to have a stronger antimicrobial activity due to a reduced particle size of 2–40 nm and an enhanced solubility in water (Bhawana et al., 2011). Accordingly, Bhawana et al. (2011) revealed a MIC of 150 mg/L of pure curcumin in DMSO and a MIC of 100 mg/L of nanocurcumin in water against *S. aureus*. In their *in vitro* study, curcumin nanoparticles exhibited antimicrobial effects against MRSA after 8 h. Colony forming unit quantification displayed a reduction of 97% in viable MRSA. Additionally, in an *in vivo* skin infection model, Krausz and colleagues investigated the antibacterial efficacy of curcumin encapsulated nanoparticles against MRSA. They stated that those nanoparticles reduce bacterial load and enhance wound healing in the mice (Krausz et al., 2015).

Photodynamic therapy has been shown to be a promising alternative therapy of infections with resistant pathogens and has been widely studied in the context of bacterial biofilm formation. In 2013, the phototoxic effect of curcumin against MRSA and MSSA was evaluated in an *in vitro* study (Ribeiro et al., 2013). Blue LED light in combination with curcumin resulted in total elimination of MSSA when curcumin was applied in concentrations of 5, 10 and 20 μM, whereas lower doses resulted in a dose-dependent decreased bacterial viability. In contrast, MRSA was only eliminated by 20 μM curcumin plus illumination, while lower concentrations still significantly reduced MRSA viability. This *in vitro* detected photokilling effect of curcumin was validated in mice by Ye et al. (2014) in a study where they analyzed the inhibitory effect of upconverted nanoparticles conjugated with curcumin (UCNPs-CCM) on MRSA-induced pneumonia. The UCNPs-CCM improved the stability and bioavailability of curcumin, ensuring better effects *in vivo* and resulting in significantly decreased bacterial counts in lungs of mice treated with UCNPs-CCM plus irradiation. The authors explained this by the fact that after irradiation, UCNPs-CCM lead to ROS production, destroying the bacterial cell membrane.

Park et al. (2005) reported an inhibitory activity of curcumin against sortase A, which is a bacterial surface protein anchoring transpeptidase. They investigated the activity of curcumin and other curcuminoids against *S. aureus* and detected an IC_50_ of 13.8 μg/ml of curcumin against sortase A, with curcumin being the most potent inhibitor among the tested curcuminoids. However, no inhibitory effect of curcumin against bacterial growth with a MIC > 200 μg/ml against *S. aureus* was observed. Inhibition of sortase A leads to a reduction of pathogenesis as shown for murine arthritis. Bacterial strains lacking sortase A are impaired in the ability to cause acute infections (Mazmanian et al., 2000; Jonsson et al., 2003). Agents targeting sortase A are thus not affecting the microbial viability but pathogenicity (Park et al., 2005).

In addition, dieacetylcurcumin, a synthetic derivative of curcumin in which two phenolic hydroxyl groups are replaced by acetyl groups, has been shown to be effective against MRSA and MSSA adhesion as well as against mature biofilm (Sardi et al., 2017).

*Staphylococcus epidermidis* is a skin commensal. Its capability of forming a biofilm on indwelling medical devices makes *S. epidermidis* a significant nosocomial pathogen. Multiple drug resistances in *S. epidermidis* have spread over the last years and the need for new agents with antimicrobial activity increased. Curcumin was shown to abrogate *S. epidermidis* growth with MICs of 10.5 to 46.9 μg/ml (Tajbakhsh et al., 2008; Liu and Huang, 2012). Aqueous extracts from *Curcuma longa* roots were also shown to act antibacterial (Niamsa and Sittiwet, 2009). Phototoxic activity of curcumin nanocarriers against *S. epidermidis* biofilms and suspensions was documented by Hegge et al. (2012): 10 μM curcumin combined with light reduced the viable cells in suspension to zero. The authors also showed that a fresh solution of supersaturated curcumin with light and without a nanocarrier kills all bacterial cells (Hegge et al., 2012).

Even though the effect of curcumin against *Staphylococci* looks promising, it has been shown that the presence of human serum albumins impedes the molecules antibacterial activity by binding it; thus hindering the molecules traverse through the bacterial membrane (Teow and Ali, 2017). *In vivo* studies or tests using human plasma or whole blood are thus needed to validate curcumin’s activity against staphylococci infections in patients.

### Curcumin Inhibits Streptococci

*Streptococcus* species are frequently found as the source of meningitis, pneumonia, and endocarditis. *S. mutans* is known for its ability to form biofilms in oral niches (Wen and Burne, 2002). However, there are also non-pathogenic *Streptococcus* species which belong to the human microbiome.

Song et al. (2012) described a suppressing activity of curcumin against the adherence of *S. mutans* to human tooth surfaces. They furthermore defined a MIC of 128 μg/ml against *S. mutans*, concentrations below the MIC diminished the adherence on both, glass surfaces as well as on human tooth. On basis of their results, the authors suggested the use of curcumin as a food-based antimicrobial agent. Also Li et al. (2018) found reduced *S. mutans* biofilm with lower extracellular polysaccharide production after treatment with curcumin in an oral habitat. In addition to pure curcumin, some studies also investigated curcumin-loaded polysaccharide nanoparticles on their antibiofilm activities against *S. mutans* in a dental model. Here, chitosan nanoparticles were revealed as the most effective form with over 75% reduction of the MIC compared to free curcumin (Maghsoudi et al., 2017). Furthermore, curcumin-PDT was shown to be effective against *S. mutans* *in vitro* (Lee et al., 2017).

Sortase A, which has been shown to be inhibited by curcumin in *S. aureus* (Park et al., 2005), is also a relevant enzyme in *S. mutans*, being responsible for covalent attachment of the major cell-surface adhesin to the cell wall, thus playing a role in biofilm formation. Intriguingly, curcumin is effective against sortase A activity and biofilm formation in *S. mutans* (Hu et al., 2013). Curcumin was also shown to act antibiostatic against *S. pyogenes* and even a synergistic effect was detected in combination with polymyxin B (Betts et al., 2016).

### Curcumin Inhibits Other Gram-Positive Bacteria

Besides *Staphylococcus* and *Streptococcus*, also other Gram-positive bacteria can be pathogenic in humans. *Bacillus* spp. are found in the human gastrointestinal tract. Free and microencapsulated curcumin showed antibiostatic activity against *B. subtilis* and *B. cereus* (Rai et al., 2008; Wang et al., 2009; Bhawana et al., 2011; Jaiswal and Mishra, 2018). While these studies found different MICs of free curcumin against *B. subtilis*, all of them showed a reduced MIC when nanocurcumin formulations were used instead (Bhawana et al., 2011; Jaiswal and Mishra, 2018). As described for *B. subtilis*, curcumin blocks the assembly and stability of FtsZ, a prokaryotic homolog of the eukaryotic cytoskeletal protein tubulin, orchestrating cell division (Rai et al., 2008). The perturbation of FtsZ functions leads to lacking bacterial proliferation, making it a suitable target for novel antimicrobial agents (Stokes et al., 2005; Kaur et al., 2010).

Another Gram-positive bacteria being a human pathogen is foodborne *Listeria*. *L. innocua* was analyzed on its susceptibility toward UVA-light exposed curcumin and a synergistic effect was detected, even when curcumin was applied in low concentrations (de Oliveira et al., 2018).

Gram-positive bacteria are considered to be less resistant against bioactive molecules and PDT than Gram-negative bacteria. This is mainly due to their outer cell wall architecture, displaying a high degree of permeability for bioactive compounds with molecular weights up to 60,000 g/mol, such as curcumin (Jori et al., 2006). Thus, Gram-negative bacteria are supposed to be more resistant against both, curcumin treatment and PDT as the outer membrane can act as a barrier for the molecules (de Oliveira et al., 2018).

### Curcumin Inhibits Gram-Negative Bacteria

Gram-negative bacteria are a large group of microorganisms, of which some can cause infections in humans. The model organism of Gram-negative bacteria is *Escherichia coli*. Shiga toxin (Stx) or Stx-like toxin producing *E. coli* is known to be an important foodborne pathogen, causing a hemolytic-uremic syndrome. In 2011, Stx-producing enterohemorrhagic *E. coli* (EHEC; serotype O104:H4) caused an outbreak in Germany affecting 3816 patients (Frank et al., 2011). Various studies demonstrated that curcumin is active against *E. coli* and the formation of its biofilms, while both effects are enhanced by curcumin nanoparticles (Wang et al., 2009; Bhawana et al., 2011; Jaiswal and Mishra, 2018). The substances activity against *E. coli* might be due to curcumin shutting of the DNA-damage response, also known as the SOS response, a complex system of genes activated, e.g., upon UV-induced mutagenesis (Oda, 1995). Another process responsible is the binding of curcumin to the bacterial FtsZ protein, decreasing cell proliferation, an effect shown for *B. subtilis* as well as for *E. coli* (Kaur et al., 2010). In addition to acting directly antibacterial, phototoxic effects of curcumin were detected against *E. coli* and the *Salmonella enterica* serotype Typhimurium already in the 1980s (Tønnesen et al., 1987; Dahl et al., 1989). Most recently, de Oliveira et al. (2018) also observed an antibacterial effect of UVA light-exposed curcumin against *E. coli* O157:H7, a mechanism which could be used to reduce cross-contamination from wash water to fresh produce.

Another important Gram-negative bacteria species is *Helicobacter pylori*, which is characterized by its ability to establish infections in the human stomach and persist there for several years. More than a half of all people worldwide are carrying *H. pylori*, causing peptic ulcer disease, gastritis and gastric cancer (Covacci et al., 1999). Because of the emergence of antibiotic resistant *H. pylori*, the need for alternative therapeutic agents is high. Curcumin was shown to be highly effective against *H. pylori* infections *in vivo* and *in vitro* (Mahady et al., 2002; De et al., 2009). De et al. (2009) showed complete eradication of *H. pylori* infection in a mouse model and even reported restoration of *H. pylori* -induced gastric damage.

Also *Pseudomonas aeruginosa*, a prominent example of a frequently multi-drug resistant organism, is a representative Gram-negative bacterium. With its ability to survive under tough environmental conditions, e.g., in antibacterial hand soaps, it marks a big issue in hospital settings (Lanini et al., 2011). *P. aeruginosa* growth is only slightly influenced by treatment with pure curcumin in a strain dependent manner (Betts et al., 2016; Jaiswal and Mishra, 2018). This effect can be boosted by the antibiotic agent polymyxin B, which alone also did not show activity against *P. aeruginosa*, but in combination with curcumin a significant synergism was detected (Betts et al., 2016). In contrast to free curcumin, curcumin-silver nanoparticles exhibit strong activity against *P. aeruginosa* and anti-biofilm activity was reported for both, pure curcumin and nanoparticles, with higher effects shown by the latter (Jaiswal and Mishra, 2018). In addition, *P. aeruginosa* growth was strongly inhibited after incubation with nano-encapsulated curcumin (Bhawana et al., 2011; Krausz et al., 2015). However, a higher activity of those nanoparticles was detected against MRSA (97% inhibition) than against *P. aeruginosa* (59% inhibition), indicating that curcumin’s effect on *P. aeruginosa* is not so strong (Krausz et al., 2015). Still, in a study on curcumin’s effects on *P. aeruginosa* virulence, a MIC of 30 μg/ml was detected, while concentrations less than the MIC still resulted in inhibition of biofilm initiation (Rudrappa and Bais, 2008). The production of virulence factors as pyocyanin was reduced and the quorum sensing system, especially acyl homoserine lactone, was also affected by curcumin. In *in vivo* models, the authors also detected that the natural compound acted preventive against *P. aeruginosa* infections of *C. elegans*: the nematode survival increased to 28% in comparison to 0% for untreated worms.

The emerging nosocomial pathogen *Stenotrophomonas maltophilia* is intrinsically resistant to β-lactams and other antibiotics with a broad spectrum (Brooke, 2012). The activity of curcumin against *S. maltophilia* was demonstrated in two *in vitro* studies (Betts et al., 2016; Yu et al., 2016). Similar as for *P. aeruginosa*, synergistic effects in combination with polymyxin B were observed (Betts et al., 2016).

## Antifungal Activities of Curcumin

Millions of fungal species can be found worldwide, but only few are human pathogens (Köhler et al., 2014; Hawksworth and Lücking, 2017). Still, fungal infections of the skin and mucosa are common, though most of them are harmless when treated. However, especially immunosuppressed individuals, such as HIV infected, cancer or organ transplant patients, are at risk of developing severe forms of infections. As with modern medicine the number of immunocompromised people increases, fungal diseases are emerging and some evolved from a rare disorder to a leading cause of illness, as observed, e.g., for cryptococcal meningitis during the HIV epidemic in Africa (Warkentien and Crum-Cianflone, 2010). In addition, global warming might contribute to a raising prevalence of fungal infections, as the geographic range of pathogenic species is increasing and they might adapt to higher temperatures, promoting their growth at body temperature (Garcia-Solache and Casadevall, 2010).

There are different types of drugs available to treat a number of fungal diseases, among them are amphotericin B, triazoles, and echinocandins (Pappas et al., 2016). Even though they are effective, severe side effects can occur and emergence of drug resistance has been observed (Klotz et al., 2016; Seufert et al., 2018). New medications are needed, especially cost-effective versions for the use in resource-limited developing countries. Turmeric has been used as a food preservative for centuries and curcumin is known to abrogate production of fungal toxins (Ferreira et al., 2013). Consequently, many studies demonstrated an antifungal effect of turmeric extracts and curcumin. In this chapter, we will recapitulate the current knowledge on curcumin’s potential to restrain the most common human pathogenic fungi. These findings are also summarized in Table 3.

**Table 3:** Antifungal activity of curcumin.

Fungi	Antifungal activity	References
*Candida* spp.	Growth inhibition Adhesion inhibition Gene expression inhibition Triggering early apoptosis	Martins et al., 2009; Shahzad et al., 2014 Sharma et al., 2010b, 2012
*Cryptococcus* spp.	Growth inhibition	Hu et al., 2017
*Aspergillus* spp.	Growth inhibition Aflatoxin production inhibition	Al-Asmari et al., 2017 Ferreira et al., 2013
Dermatophytes	Growth inhibition	Brasch et al., 2017, 2018


### Curcumin Inhibits *Candida* spp.

*Candida* spp., a genus of yeast, is a commensal fungus usually found on the skin and on the mucosa of the gastrointestinal tract and mouth. It is the most common cause of fungal infections in humans, as it can elicit opportunistic infections known as candidiasis. This can affect different parts of the body, but the most frequent forms are oropharyngeal or vulvovaginal candidiasis. *Candida* spp. can become invasive and lead to systemic infections of the blood, candidemia, or to deep-seated tissue candidiasis. It still is the most common fungal disease among hospitalized patients in the developed world, causing more than 50,000 deaths per year (Kullberg and Arendrup, 2015).

A prerequisite for candidiasis is the adhesion of *Candida* to human cell surfaces. Intriguingly, curcumin has been shown to block the adhesion of *Candida* spp. to buccal epithelial cells (Martins et al., 2009) and the development of *C. albicans* biofilms by downregulating adhesin and filamentation-associated genes (Shahzad et al., 2014). In general, curcumin exhibits antifungal activity against planktonic forms of standard and at least 10 clinical *Candida* strains (Neelofar et al., 2011). Sharma et al. (2010b, 2012) explained this by curcumin-induced modifications of the fungus membrane lipid composition, which eventually leads to the production of ROS, triggering early apoptosis. Furthermore, Kumar et al. (2014) showed that curcumin downregulates cell wall integrity pathway genes, causing cell wall damage and membrane permeabilization in *C. albicans*.

Upon infection, candidiasis due to *C. albicans* is often treated with the antifungal drug fluconazole (Pappas et al., 2016). Several studies showed that curcumin is far more potent than fluconazole (Martins et al., 2009), acts synergistic with the drug (Sharma et al., 2010a) and even restores sensitivity to it in resistant *C. albicans* clinical isolates (Garcia-Gomes et al., 2012). This might be due to curcumin’s ability to modulate the activity of ABC and MDR transporters, which facilitate the active efflux of multiple drugs in resistant strains (Sharma et al., 2009; Garcia-Gomes et al., 2012).

Due to the development of drug resistance, alternative therapy options are needed. In this context, several studies examined curcumin’s potential as a sensitizer for photodynamic inactivation of fungi and found that, *in vitro*, it indeed inactivated or at least inhibited the growth of different *Candida* strains and isolates (Dovigo et al., 2011; Andrade et al., 2013; Sanitá et al., 2018). Dovigo et al. (2013) also used curcumin as a photosensitizer in a murine model of oral candidiasis, where it drastically reduced colony counts. Curcumin’s photosensitizing activity might be explained by a genotoxic effect, as the compound seems to prevent repair of DNA damage caused by light (Carmello et al., 2015), similar as observed for *E. coli* (Oda, 1995). Another non-invasive treatment option is the local application of curcumin containing formulations. A recent study used a cream containing 1.0% curcumin to treat vulvovaginal candidiasis in a rat model (Fernandes et al., 2018). The authors observed a reduction of fungal burden and inflammatory infiltration due to the cream. Therefore, curcumin formulations could be a promising alternative to combat candidiasis.

### Curcumin Inhibits *Cryptococcus* spp.

*Cryptococcus* is a widespread encapsulated yeast, and some of its species, including *C. neoformans*, *C. gattii* and *C. bacillispores*, are the causative agents of the most common invasive fungal infections in humans, called cryptococcosis. Cryptococcal infections affect mainly immunocompromised patients and show high mortality and morbidity rates. As treatment options are limited and resistance emerges, new therapeutic options are needed.

A recent study found that in *C. neoformans*, curcumin accumulates in the endoplasmic reticulum, causing a growth-reduction (Hu et al., 2017). The authors showed that this is probably due to curcumin’s iron-chelator activity. In line with this, another study found that, in mice, curcumin alone or in combination with fluconazole significantly reduces pulmonary damage and fungal burden of *C. gattii* infections (da Silva et al., 2016). Further research is needed to reveal the exact mechanisms of action and to show whether curcumin holds the potential to be a new drug option to cure cryptococcosis.

### Curcumin Inhibits *Aspergillus* spp.

The genus *Aspergillus* contains over a 100 mold species, of which *A. fumigatus* causes the most invasive infections in humans (Schmitt et al., 1990). Aspergillosis is a group of diseases, including, for example, non-invasive infections of the respiratory tract, the ears or the eyes. After major surgery or upon immunosuppression, patients sometimes develop severe invasive and potentially lethal forms of aspergillosis (Zilberberg et al., 2018). Besides causing ailment, *Aspergillus* is known for contaminating improperly stored food. As some strains produce aflatoxins, consumption of spoiled food often leads to poisoning, which in turn can cause hepatic injury (Probst et al., 2007; Yard et al., 2013).

Several studies showed that *Aspergillus* isolates were not affected by curcumin essential oil (Tantaoui-Elaraki and Beraoud, 1994) and that pure curcumin only reduced fungal growth in very high concentrations of over 256 mg/L (Martins et al., 2009). However, even though the natural compound does not directly act antifungal on *Aspergillus*, it was shown to reduce aflatoxin production: already 0.1% curcumin abrogated the production of the toxin in *A. flavus* (Ferreira et al., 2013). In addition, curcumin seems to act beneficial on aflatoxin-induced liver and kidney injury in mice and chicken (Verma et al., 2008; Zhang et al., 2016), probably by reducing aflatoxin-mediated oxidative stress in a dose-dependent manner (Wang et al., 2018).

Recent studies also examined curcumin’s photosensitizing ability in PDT of *Aspergillus* infections. Similar as for other fungi, curcumin significantly inhibited the growth of cells and spores of *A. flavus* and *A. niger* (Al-Asmari et al., 2017).

### Curcumin Inhibits Dermatophytes

Dermatophytes is a group of fungi consisting of over 40 species in the genera of *Microsporum*, *Epidermophyton*, and *Trichophyton*. All of them commonly cause skin infections, called tinea or dermatophytosis, like athlete’s foot (tinea pedis).

Early studies indicated that *Curcuma longa* L. oils, but not curcumin itself, act against dermatophytosis caused by *Trichophyton* in guinea pigs (Apisariyakul et al., 1995). In contrast, another study found that volatile oils extracted from turmeric, consisting of at least 10% curcumin, dampened the growth of 29 clinical dermatophytes strains *in vitro* using an agar disk diffusion method (Wuthi-udomlert et al., 2000). Recent studies investigated curcumin’s potential as a photosensitizer in dermatophytosis treatment. Brasch et al. (2017, 2018) found that curcumin plus visible light significantly inhibited the conidia- and mycelial-derived growth of different dermatophytes species for at least 96 h. Thus, the development of a PDT with curcumin against dermatophytosis could be a promising novel therapeutic option.

## Obstacles and Future Perspective of Curcumin as an Anti-Infective Agent

Comprehensive clinical trials evaluated the safety, pharmacokinetics and effectiveness of curcumin against different diseases (as reviewed, e.g., Goel et al., 2008). Despite its excellent tolerability with no or minimal toxicity even at high oral doses of up to 12 g/day, its poor bioavailability leads to only low serum concentrations, limiting the exploitation of its potentially valuable therapeutic effects (Cheng et al., 2001; Lao et al., 2006). Curcumin’s low bioavailability can be explained by its insolubility in water at neutral pH and the facts that it degrades or crystallizes in alkaline and acidic solutions, respectively (Tønnesen and Karlsen, 1985; Kharat et al., 2017). In human blood curcumin is more stable with a half-life of approximately 8 h (Wang et al., 1997). However, the major part of orally administered curcumin never reaches the blood, as it is poorly absorbed from the intestine and directly discharged again (Holder et al., 1978; Wahlström and Blennow, 1978). In addition, curcumin taken up into the blood is rapidly metabolized, conjugated in the liver and excreted in the feces (Holder et al., 1978; Ravindranath and Chandrasekhara, 1981-1982), therefore it has limited systemic bioavailability.

**FIGURE 2:**
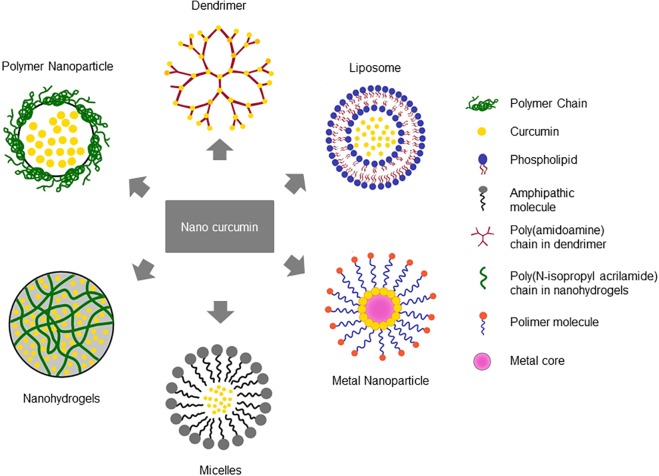
Nanocarrier systems encapsulating curcumin. Different nanoformulation strategies have been reported to improve the bioavailability and therapeutic efficacy of curcumin based on their chemical composition.

To overcome these drawbacks, several nanoparticle-based approaches have been developed. In general, there are two proposed nanoforms for curcumin: nanocrystals, and nanocarriers. Nanocrystals are particles with nanometer-range size, which have been engineered as a pharmacological tool for drugs with limited solubility. Due to their small size, the molecules surface area and solubility are increased, leading to an enhanced dissolution rate and bioavailability, as e.g., shown for curcumin combined with different stabilizing agents (Rachmawati et al., 2013). However, nanocrystals do not solve the problems of the pre-systemic degradation and rapid systemic metabolism of curcumin. Thus, different nanocarrier systems encapsulating the natural compound have been developed. These include, among others, curcumin incorporated within polymer nanoparticles or nanovesicles such as liposomes or micelles; matrix-based formulations such as hydrogels and nanoemulsions. Exemplarily, some nanocarrier-curcumin systems are depicted in Figure 2. Their advantages compared to pure curcumin are given in Table 4. Curcumin-loaded nanocarrier systems do not only show enhanced solubility, uptake and bioavailability compared to the pure substance, but they also protect it from external and internal degradation or early metabolism. But still, after entering the body, the carriers are rapidly taken up by the liver and spleen, leading to a relatively short circulation time. Moreover, e.g., nanoemulsions also contain multiple surfactants, which can lead to toxicity. However, due to the great potential benefit in therapy, the development and refining of curcumin-nanocarrier formulations for the treatment of various diseases is still ongoing.

**Table 4:** Formulations of curcumin to improve its bioavailability.

Formulation	Effect	References
Polymer nanoparticle	Improved stability and solubility, enhanced antibacterial effect	Pietra et al., 2017; Trigo Gutierrez et al., 2017
Liposome	Increased bioavailability	Chen et al., 2009
Micelle	Solubility and antibacterial activity	Liu and Huang, 2012
Dendrimer	Improved solubility	Falconieri et al., 2016
Nanogel	Improved solubility and bioavailability	Reeves et al., 2015
Metal nanoparticle	Improved stability and solubility	Sindhu et al., 2014


## Conclusion

Numerous *in vitro* and *in vivo* studies have shown that curcumin is active against different viruses, bacteria and fungi, including even highly pathogenic, emerging and multi-drug-resistant strains. The underlying mechanism seems to be complex and to differ from organism to organism, thus it has not always been elucidated. As curcumin is not toxic even at high oral doses and as it is already approved and widely used in the food industry, its broad-spectrum anti-infective activity makes it a promising drug candidate. Unfortunately, the molecule’s poor solubility, low bioavailability, and rapid metabolism hamper its use in clinical settings and resulted in no observable therapeutic effects in many clinical trials. However, it should be kept in mind that most clinical trials were analyzing systemic applications of curcumin and were focused on general safety aspects or on the treatment of cancer; studies of curcumin’s systemic activity against infectious diseases in humans are largely missing. Nevertheless, clinical assessment showed that the topical oral or cervical application of curcumin had no toxic effect and led to the disaggregation of oral plaque (Leite et al., 2014) and to an enhanced HPV clearance (Basu et al., 2013; Gattoc et al., 2017), respectively. This suggests that at least the local treatment with curcumin is effective against bacteria and viruses in humans. The development of curcumin formulations in various nanocarrier systems could help to overcome the obstacles experienced in systemic curcumin application, paving the way to new (infectious disease) clinical trials with the natural product.

Concluding, further research is required to fully understand curcumin’s mode of action and to improve formulations to make it usable as a drug. Clinical trials will then show whether its effect seen in the lab will hold true in patients.

## Author Contributions

DP, JB, and ES contributed to Antiviral section. LK and JS contributed to Antibacterial and Antifungal section. HR contributed to Obstacles and Future Perspective of Curcumin as an Anti-Infective Agent section.

## Conflict of Interest Statement

The authors declare that the research was conducted in the absence of any commercial or financial relationships that could be construed as a potential conflict of interest.
